# Data-Driven Quantification of Quantum *k*-Entanglement via Machine Learning

**DOI:** 10.3390/e28070832

**Published:** 2026-07-22

**Authors:** Jie Guo, Jinchuan Hou, Xiaofei Qi, Kan He

**Affiliations:** 1 College of Mathematics, Taiyuan University of Technology, Taiyuan 030024, China; jieguo11@163.com; 2 School of Mathematical Mathematics and Statistics, Shanxi University, Taiyuan 030006, China; 3 Key Laboratory of Complex Systems and Data Science of Ministry of Education, Shanxi University, Taiyuan 030006, China

**Keywords:** multipartite systems, entanglement states, machine learning, *k*-nonseparability, *k*-entanglement measures

## Abstract

*k*-entanglement, including entanglement relative to full separability and genuinely multipartite entanglement, is a fundamental quantum resource in multipartite quantum systems. Its identification and quantification play essential roles in quantum information processing, quantum simulation, and quantum metrology. However, the practical computation of rigorous *k*-entanglement measures remains highly challenging due to the need for high-dimensional optimization. In this work, we propose a machine-learning-based surrogate framework for approximating the witness-based *k*-entanglement measure $E_{w}^{\left(k,n\right)}$. The numerical evaluation of the computationally realized quantity ${\tilde{E}}_{w}^{\left(k,n\right)}\left(\rho \right)$ is reformulated as a supervised regression problem, where the input is the density matrix ρ and the labels are obtained from finite witness databases. The framework combines multilayer perceptrons (MLPs), convolutional neural networks (CNNs), and light gradient boosting machine (LightGBM) through a stacking ensemble. Numerical experiments are performed for 3- and 4-qubit systems as representative demonstrations of the proposed workflow. The results show that the learned models achieve high predictive accuracy in terms of MAE, MSE, and $R^{2}$, while providing millisecond-level inference for single-state evaluation. Werner state tests serve as symmetric benchmark checks, and an additional four-qubit noisy circuit-generated state family, obtained from finite-depth circuit preparation followed by local amplitude-damping noise, is used as a structured physical test beyond random density matrices. Compared with the optimization-based evaluation, the trained surrogate model significantly reduces the computational time while maintaining accuracy within the tested system sizes and data distributions. These results show that the proposed framework provides an efficient numerical surrogate for rapid approximation of witness-based *k*-entanglement measures, while extensions to larger systems and experimental data require further validation.

## 1. Introduction

Quantum entanglement, as one of the most essential resources in quantum information science, plays a foundational role across quantum computing, quantum communication, quantum simulation, and quantum metrology. Its nonclassical correlations not only underpin the mechanisms enabling quantum algorithmic speed-ups and the formulation of quantum computational models, but also constitute the physical basis for the security of quantum communication protocols. Likewise, high-precision quantum simulation and measurement schemes that surpass classical limits critically rely on the resource characteristics provided by entanglement. Consequently, from fundamental theory to practical engineering, entanglement occupies an irreplaceable position within the entire quantum technology stack [1,2,3,4,5,6,7,8,9,10].

Practically, one often treats with entanglement in multipartite quantum systems. However, the structure of entanglement in multipartite quantum systems is substantially more intricate than in bipartite scenarios, exhibiting an explicit hierarchical organization as the number of subsystems and admissible partition patterns increase. The classification framework based on *k*-separability and *k*-entanglement enables a systematic, layer-wise characterization of multipartite entanglement according to subsystem partitions, thereby revealing the organization of correlations at different structural scales [11,12,13,14]. Such hierarchical descriptions offer a more discriminative perspective on the resource properties of multipartite entanglement and are closely linked to quantum resource availability, the expected performance of scalable quantum algorithms, and the controllability of complex multipartite states. Hence, achieving fast and accurate quantification of *k*-entanglement in multipartite systems has become a key requirement for advancing both quantum information processing and the engineering of large-scale quantum systems.

For an *n*-partite system and 2≤k≤n, the *k*-entanglement, including multipartite entanglement (k=n) and genuinely multipartite entanglement (k=2), is a crucial quantum resource in multipartite systems. Its identification and quantification are fundamental tasks in quantum information theory. The corresponding measures quantify the degree of *k*-entanglement and satisfy the axiomatic constraints imposed by quantum mechanics and many-body resource theory. However, recognizing *k*-entanglement is NP-hard, and to date, the practical utility of existing rigorous quantifications of *k*-entanglement for general multipartite states remains limited to theoretical analysis. These quantifications are not directly computable, even uncomputable, as high-dimensional optimization, spectral decompositions, exhaustive enumeration over subsystem partitions, and convex roof extension make the computations nearly impractical. In particular, the computational cost grows exponentially with system dimension, making even low-dimensional systems challenging in practice [15,16,17,18,19,20]. In response to these challenges, a *k*-entanglement measure $E_{w}^{\left(k,n\right)}$ for arbitrary *n*-partite systems, meeting the requirements of many-body resource theory, has been proposed in [21]. It also provided a mathematically rigorous solution of how to evaluate $E_{w}^{\left(k,n\right)}$ and a method of constructing a computation software tool based on specially created datasets of *k*-entanglement witnesses. Thus the approach in [21] solves essentially the problem of detecting the *k*-entanglement degree of arbitrary multipartite states. However, although the method for computing $E_{w}^{\left(k,n\right)}\left(\rho \right)$ at any state ρ is universal, formally exact and its value can be obtained by a pre-established software tool based on suitably constructed datasets of *k*-entanglement witnesses, the preparation of such datasets requires traversing all subsystem partitions and performing large-scale matrix optimizations. This results in high computational overhead in practice when dimensions are large. Another issue is that, even if the calculation software has been devloped, to obtain the value of $E_{w}^{\left(k,n\right)}\left(\rho \right)$ for any given state ρ, running the software still takes considerable time, which is fundamentally incompatible with the dynamical timescales of current quantum platforms and limits the practical application of the detection software. Whether in superconducting circuits, trapped-ion systems, or photonic architectures, the coherence time of multipartite entangled states typically lies in the microsecond-to-millisecond regime, whereas even for four-qubit states, a full evaluation of measure $E_{w,4}^{\left(k,n\right)}\left(\rho \right)$ may require hundreds of seconds or more [21], and the time required will grow exponentially with the increase in the number of qubits. This timescale mismatch makes real-time entanglement monitoring, online feedback control, and continuous parameter scanning exceedingly difficult in experiments, forming a major bottleneck that hinders the transition of multipartite entanglement from theoretically solvable to experimentally deployable. In this work, we mainly focus on the latter issue and try to address it using machine learning methods.

Benefiting from powerful feature-extraction capabilities and highly expressive nonlinear modeling, machine learning has shown notable success in quantum communication [22,23,24], quantum computation [25,26], quantum optics [27], quantum state tomography [28], quantum metrology [29], and quantum-correlation detection [30,31,32,33,34,35,36,37,38,39,40,41,42,43,44,45,46,47].While machine learning has achieved success in classification tasks, its application to quantifying quantum correlations remains relatively limited [48,49]. Existing machine-learning-based studies have mainly focused on bipartite entanglement detection and lower-bound estimation of certain entanglement measures. Generally, entanglement identification is treated as a classification task, while regression-based approaches aim to predict numerical quantities related to entanglement. However, most entanglement measures are defined through highly nonlinear optimization problems over high-dimensional quantum state spaces. As a consequence, exact values of entanglement measures are generally inaccessible as supervision during training, and current models rely instead on computable lower bounds or surrogate quantities derived from entanglement witnesses, semidefinite relaxations, or PPT-based criteria. Though effective for entanglement certification, such quantities only provide conservative estimates and do not capture the full quantitative structure of entanglement across the state space. This issue is further amplified in experimental scenarios, where limited measurement resources restrict direct access to exact entanglement measures. Moreover, these limitations become particularly pronounced when quantifying *k*-entanglement in multipartite systems, and no studies have yet applied machine learning methods to quantify *k*-entanglement. This leads to the central question of this work: *How data-driven models can learn the high-dimensional, nonlinear mapping between a density matrix ρ and its rigorous k-entanglement measures, thereby compressing an exponentially complex optimization into a single fast forward pass while maintaining physical reliability?*

To address this question, in this work, we choose the *k*-entanglement measure $E_{w}^{\left(k,n\right)}$ from [21] and develop a machine-learning-based strategy for quantifying *k*-entanglement in finite-dimensional multipartite quantum systems. The rigorous optimization process is reformulated as a supervised regression task that approximates the functional mapping $\rho \mapsto E_{w}^{\left(k,n\right)}\left(\rho \right)$. During training, the computationally realized value ${\tilde{E}}_{w}^{\left(k,n\right)}$ obtained from the finite witness database is used as the supervision signal, with Hermitian, trace-one, positive semidefinite density matrices as input. Numerical tests show that, after training, the model performs inference in milliseconds, achieving a speed-up of more than five orders of magnitude relative to the exact solver and bridging the long-standing gap between theoretical precision and experimental real-time requirements.

To balance accuracy, robustness, and generalization, we construct a heterogeneous ensemble comprising multilayer perceptron (MLP), convolutional neural network (CNN), and light gradient boosting machine (LightGBM) models. Through stacking ensemble techniques, the complementary strengths of the three architectures are effectively integrated: MLP captures global nonlinear dependencies, CNN provides a parameter-sharing representation-learning mechanism for matrix-valued inputs under a fixed basis ordering, and LightGBM excels in high-dimensional feature selection and interpretable regression [50,51,52,53,54]. This integration of heterogeneous models markedly improves performance across varying system sizes and entanglement levels. With inference achievable at millisecond scales, the approach supports real-time entanglement monitoring and experiment-side feedback control, offering a computationally efficient surrogate approach for fixed-system *k*-entanglement quantification, with potential relevance to future experimental workflows after platform-specific validation for efficient quantification of high-order entanglement in multipartite systems.

The structure of this paper is organized as follows. Section 2 introduces the essential preliminaries for the subsequent study, including the definition of *k*-entanglement in multipartite systems, associated criteria, and the *k*-entanglement measure $E_{w}^{\left(k,n\right)}$ for *n*-partite systems with 2≤k≤n, which form the basis for constructing the labels used in our dataset. Section 3 establishes a systematic methodological route for fast numerical approximation of *k*-entanglement by presenting a general machine learning surrogate framework, covering the design of base models, the stacking ensemble architecture, the dataset generation strategy, and the performance evaluation procedure. Section 4 reports the numerical results and assesses the model’s accuracy, efficiency, and behavior within the tested distributions. Using three-qubit and four-qubit systems, Werner states, and a four-qubit noisy circuit-generated state family as examples, we examine the agreement and differences between machine learning predictions and witness-based numerical values. Section 5 gives a short conclusion.

## 2. The k-Entanglement Measure $E_{w}^{\left(k,n\right)}$

Before turning to our main analysis, it is necessary to consolidate the basic notions, separability hierarchies, and quantitative tools underlying the characterization of multipartite quantum entanglement. In particular, we recall the definitions of *k*-separability and *k*-entanglement, elucidate the nested structural relations between different separability classes, and outline an entanglement-witness-based framework that enables rigorous quantification of multipartite correlations.

The *k*-entanglement exhibits a hierarchical structure of nonclassical correlations distributed across different collections of subsystems. We first recall that a quantum system can be described by a complex separable Hilbert space *H*, and quantum states are described as density operators, namely positive trace-one operators, on *H*. A pure state ρ is a rank-one projection; that is, the dimension of its range is one, and it can be written as ρ=|ψ〉〈ψ| for some unit vector |ψ〉∈H. A state that is not pure is called a mixed state. For convenience, we also refer to a unit vector |ψ〉∈H as a pure state. For any positive integer *n*, an *n*-partite quantum system *H* is given by the tensor product of *n* subsystems, i.e., $H=H_{1}⊗H_{2}⊗\cdots ⊗H_{n}.$ Let $S(H_{1}⊗\cdots ⊗H_{n})$ denote the set of all *n*-partite states on $H=H_{1}⊗\cdots ⊗H_{n}$, and let $Pur(H_{j})$ denote the set of pure states on $H_{j}$. A state ρ is fully separable if

(1) $$\rho =\sum_{r=1}^{h}p_{r}\rho_{1,r}⊗\cdots ⊗\rho_{n,r}$$

for $\rho_{j,r}\in Pur\left(H_{j}\right)$ and $p_{r}\geq 0$ with $\sum_{r=1}^{h}p_{r}=1$, or if it is a trace-norm limit of such finite convex combinations when dimH=∞. Here *h* denotes the number of product pure-state terms in a finite convex decomposition, and the same convention will be used below. Such a decomposition is generally not unique. In the finite-dimensional case, let $d_{j}=\dim H_{j}$ and $D=\dim H=\prod_{j=1}^{n}d_{j}$. Since the density operators on *H* are contained in the real affine space of Hermitian trace-one operators of dimension $D^{2}-1$, Carathéodory’s theorem implies that every fully separable state can be represented as a convex combination of at most $D^{2}$ product pure states. Thus, without loss of generality, one may take $h\leq D^{2}$ in finite dimensions. If all local dimensions are equal to *d*, this bound becomes $h\leq d^{2n}$. In infinite-dimensional systems, fully separable states are understood as trace-norm limits of finite convex combinations of product pure states; hence no universal finite upper bound on *h* is available, and *h* refers only to the number of terms in each finite approximating decomposition. For bipartite systems (n=2), the usual separability coincides with full separability. For n>2, it may happen that some subsystems are separable while others are entangled. This motivates the concept of *k*-separability. For k≤n, a *k*-partition $P=P_{1}|P_{2}\left|\cdots \right|P_{k}$ of 1,2,…,n means that $P_{j}\subseteq 1,2,\ldots ,n$, $P_{i}\cap P_{j}=\emptyset$ for i≠j, and ${⋃}_{j=1}^{k}P_{j}=1,2,\ldots ,n$. Every *k*-partition *P* determines a *k*-partite decomposition $H=H_{P_{1}}⊗H_{P_{2}}⊗\cdots ⊗H_{P_{k}},$ where $H_{P_{j}}={⨂}_{s\in P_{j}}H_{s}.$ Clearly, we have $H_{P}=H$. For 2≤k≤n, a pure state $|\psi \rangle \in H_{1}⊗\cdots ⊗H_{n}$ is said to be *k*-separable if there exists a *k*-partition $P=P_{1}|P_{2}\left|\cdots \right|P_{k}$ of 1,2,…,n such that $\left|\psi \rangle =\right|\psi_{P_{1}}\rangle ⊗|\psi_{P_{2}}\rangle ⊗\cdots ⊗|\psi_{P_{k}}\rangle ,$ where $|\psi_{P_{j}}\rangle \in H_{P_{j}}$, j=1,2,…,k. A mixed state $\rho \in S(H_{1}⊗\cdots ⊗H_{n})$ is *k*-separable if it is a convex combination

$$\rho =\sum_{r=1}^{h}p_{r}|\psi_{r}\rangle \langle \psi_{r}|$$

of *k*-separable pure states $|\psi_{r}\rangle$, with the same convention for *h* as above, or if it is a trace-norm limit of such finite convex combinations in the infinite-dimensional case; otherwise, it is called *k*-nonseparable or *k*-entangled. It is important to note that the above *k*-separable pure states $|\psi_{r}\rangle$ may depend on different *k*-partitions. Clearly, ρ is *n*-separable if and only if it is fully separable, and is genuinely multipartite entangled if and only if it is 2-entangled. These two important kinds of multipartite entanglement have been studied intensively and extensively. Let $S_{k}=S_{k}\left(H_{1}⊗\cdots ⊗H_{n}\right)$ denote the set of all *k*-separable states. For each *k* with 2≤k≤n, $S_{k}$ is a closed convex subset of $S(H_{1}⊗\cdots ⊗H_{n})$, and the sets form a strict hierarchy,

$$S_{n}\subset S_{n-1}\subset \cdots \subset S_{2}.$$

A state ρ is *k*-entangled if and only if $\rho \notin S_{k}$.

An approach to quantify the *k*-entanglement is achieved by using *k*-entanglement witnesses [12,55]. A Hermitian operator $W\in B(H_{1}⊗\cdots ⊗H_{n})$ is a *k*-entanglement witness if 〈ψ|W|ψ〉≥0 for all *k*-separable pure states |ψ〉 and *W* is not positive semidefinite. Here, as usual, B(H) denotes the set of all bounded linear operator acting on *H*. A state ρ is *k*-entangled if and only if there exists such a witness with $\mathrm{Tr}(W_{k}\rho )<0$. Since the convex geometry of $S_{k}$ typically exhibits highly nonlinear boundaries, entanglement witnesses act as separating hyperplanes, providing an operational and geometric foundation for defining continuous *k*-entanglement measures. Building on this perspective, a rigorous witness-based quantitative measure for *k*-entanglement is introduced in [21]. Let $P_{n}^{k}$ be the set of all *k*-partition $P=P_{1}|P_{2}\left|\ldots \right|P_{k}$ of 1|2|…|n and

(2) $$B_{1}^{+}=\{L\in B\left(H_{1}⊗\cdots ⊗H_{n}\right):\;L\geq 0,\;∥L∥\leq 1\}.$$

For $P=P_{1}|P_{2}\left|\ldots \right|P_{k}\in P_{n}^{k}$, let

(3) $$\begin{array}{cc} g(L,P)= & \sup\{\langle \phi_{P_{1}}\phi_{P_{2}}\ldots \phi_{P_{k}}\;|\;L\;|\;\phi_{P_{1}}\phi_{P_{2}}\ldots \phi_{P_{k}}\rangle \;:\;|\phi_{P_{j}}\rangle \in H_{P_{j}},\\ \; & \;\;\;\langle \phi_{P_{j}}|\phi_{P_{j}}\rangle =1,\;j=1,\ldots ,k\}. \end{array}$$

and define

(4) $${\mathrm{EW}}_{1}^{\left(k\right)}=\{\lambda I-L:L\in B_{1}^{+},\;g_{n}^{\left(k\right)}\left(L\right)\leq \lambda <∥L∥\},$$

where

(5) $$g_{n}^{\left(k\right)}\left(L\right)=\max_{P\in P_{n}^{k}}g\left(L,P\right).$$

The *k*-entanglement measure $E_{w}^{\left(k,n\right)}$ proposed in [21] is defined as

(6) $$E_{w}^{\left(k,n\right)}\left(\rho \right)=\underset{W_{k}\in {\mathrm{EW}}_{1}^{\left(k\right)}}{\sup}\left|\min\{\mathrm{Tr}\left(W_{k}\rho \right),0\}\right|=\underset{\begin{array}{c} L\in B_{1}^{+} \end{array}}{\sup}\max\left\{\mathrm{Tr}\left(L\rho \right)-g_{n}^{\left(k\right)}\left(L\right),0\right\}$$

for any $\rho \in S(H_{1}⊗\cdots ⊗H_{n})$. The measure $E_{w}^{\left(k,n\right)}$ introduced in [21] is nonnegative, convex, sub-additive, monotonic under LOCCs, and invariant under subsystem permutations, and it satisfies the axioms of many-body resource theory, including the condition that the *k*-entanglement contained in subsystems does not exceed that of the whole system. In particular, $E_{w}^{\left(k,n\right)}$ vanishes exactly on *k*-separable states, providing a quantitative characterization of multipartite *k*-entanglement.

A remarkable property of $E_{w}^{\left(k,n\right)}$ is its computability. To compute this measure in practice, Ref. [21] gave an algorithm for g(L,P) in Equation (3) and proposed a numerical scheme based on sampling positive operators $L\in B_{1}^{+}$ and evaluating their extremal expectations $g_{n}^{\left(k\right)}\left(L\right)$ across all relevant partitions. The resulting database

(7) $$\begin{array}{cc} & \tilde{\mathrm{EW}}\left(H_{1}⊗H_{2}⊗\cdots ⊗H_{n}\right)\\ = & \{[L,g\left(L\right),\left\{g\left(L,P\right):P\in P^{\left(n-1\right)}\right\},\ldots ,\left\{g\left(L,P\right):P\in P^{3}\right\},\\ & \left\{g\left(L,P\right):P\in P^{2}\right]:L\in {\tilde{B}}_{1}^{+}\} \end{array}$$

with ${\tilde{B}}_{1}^{+}$ a finite subset of $B_{1}^{+}\left(H_{1}⊗H_{2}⊗\cdots ⊗H_{n}\right)$ enables the approximation

(8) $${\tilde{E}}_{w}^{\left(k,n\right)}\left(\rho \right)=\max_{L\in {\tilde{B}}_{1}^{+}}\max\left\{\mathrm{Tr}\left(L\rho \right)-\max_{P\in P_{n}^{k}}g\left(L,P\right),0\right\}$$

of $E_{w}^{\left(k,n\right)}\left(\rho \right)$ for each k∈{2,3,…,n} and any state ρ, which converges to the true value $E_{w}^{\left(k,n\right)}\left(\rho \right)$ as the sampling set becomes sufficiently dense. Although fully computable whenever $\tilde{\mathrm{EW}}$ is prepared and the accuracy of approximation is sufficiently high, the procedure of computing approximation ${\tilde{E}}_{w}^{\left(k,n\right)}\left(\rho \right)$ of $E_{w}^{\left(k,n\right)}\left(\rho \right)$ in Equation (8) still becomes increasingly demanding with the system size due to the exponential number of partitions. Even for small *n*, for instance, considering *m*-qubit system with n≤m≤4, exact computation of Equation (8) typically requires hundreds of seconds. Evaluating the 3-entanglement measure for a four-qubit system, with sample number 30,000 in ${\tilde{B}}_{1}$, requires roughly 200 s per state, highlighting the intrinsic theoretical difficulty of quantifying multipartite *k*-entanglement and the conflict with experimental constraints. In superconducting, trapped-ion, and photonic quantum platforms, entangled states typically maintain coherence only on the microsecond-to-millisecond timescale. Exact evaluation of the measure using the software tool developed in [21] is therefore impractical, making real-time monitoring, online feedback, and adaptive control infeasible.

To circumvent this computational-time bottleneck, we utilize the highly accurate approximation ${\tilde{E}}_{w}^{\left(k,n\right)}\left(\rho \right)$ serving as supervised labels for machine learning models trained to learn the nonlinear map $\rho \mapsto E_{w}^{\left(k,n\right)}\left(\rho \right)$. Once trained, such models replace the expensive optimization with a single forward inference, preserving the rigorous physical meaning of the measure while providing substantial gains in computational efficiency. This approach furnishes a scalable and physically grounded route for quantifying multipartite *k*-entanglement in high-dimensional quantum systems.

## 3. Training Process of the Machine Learning Models

This section presents a data-driven framework for approximating the multipartite *k*-entanglement measure $E_{w}^{\left(k,n\right)}\left(\rho \right)$ in finite-dimensional systems. The objective is to learn a direct mapping from the density matrix to the target quantity based on physically valid training data.

Neural networks, supported by the Universal Approximation Theorem [56,57], provide flexible nonlinear function approximation, while tree-based ensemble methods such as LightGBM model feature interactions through gradient boosting over decision trees. These complementary modeling strategies enable effective representation of high-dimensional nonlinear relationships. All models are trained on datasets consisting of density matrices satisfying Hermiticity, unit trace, and positive semidefiniteness. Once trained, the models estimate $E_{w}^{\left(k,n\right)}\left(\rho \right)$ directly from the input density matrix, avoiding explicit numerical optimization of Equation (8) and reducing computational cost at inference. To accommodate the structural complexity of density matrices, a hierarchical ensemble framework is adopted. At the base level, MLP, CNN, and LightGBM are employed to capture global nonlinear mappings, localized structural patterns, and feature interactions, respectively. At the ensemble level, a stacking strategy combines the out-of-fold predictions of the base learners through a meta-learner, yielding a two-stage predictive mapping from the density matrix to the estimated *k*-entanglement value. The training procedure consists of optimizing the base learners followed by fitting the meta-learner. Inference requires only forward evaluation of the trained models.

The following subsections describe the base model configurations, the stacking mechanism, dataset construction and preprocessing, and the evaluation protocol, which together define the proposed machine learning framework.

### 3.1. Base Model Design

At the base level of the hierarchical ensemble framework, three models, MLP, CNN, and LightGBM, are employed. These models rely on different inductive biases and learning mechanisms, thereby providing complementary representations of the input density matrix. Their combination establishes a diversified feature learning stage that supports the subsequent stacking aggregation.

**Figure 1:**
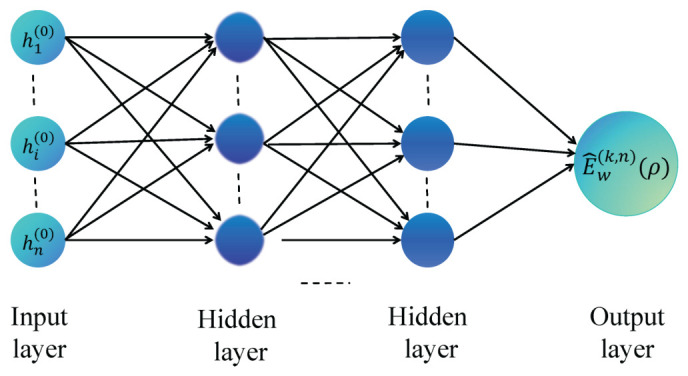
Illustration of the multilayer perceptron (MLP) architecture. The input feature vector h, derived from the unfolded density matrix ρ, passes through multiple hidden layers with nonlinear ReLU activations to learn the global and high-dimensional nonlinear characteristics of the quantum state. The output layer produces the predicted *k*-entanglement measure ${\hat{E}}_{w}^{\left(k,n\right)}\left(\rho \right)$.

**(a) MLP.** The MLP is a class of feedforward neural networks whose core idea is to learn complex mappings from input features to target outputs through a series of linear transformations followed by nonlinear activations. In this work, the input to the MLP is the flattened feature vector of the quantum density matrix ρ, which includes the real parts of the diagonal elements and both the real and imaginary parts of the upper-triangular elements. Each hidden layer performs a linear transformation followed by a nonlinear activation, which can be formally expressed as

(9) $$h^{\left(l\right)}=f^{\left(l\right)}\left(W^{\left(l\right)}h^{\left(l-1\right)}+b^{\left(l\right)}\right),\;l=1,2,\ldots ,L,$$

where $h^{\left(0\right)}$ denotes the input feature vector, $W^{\left(l\right)}$ and $b^{\left(l\right)}$ are the weight matrix and bias vector of the *l*-th layer, respectively, and $f^{\left(l\right)}\left(\cdot \right)$ represents the activation function. In this study, the rectified linear unit (ReLU) activation function f(x)=max(0,x) is adopted to mitigate the vanishing gradient problem and to improve numerical stability during training. The final network output, denoted as $\hat{E}=h^{\left(L\right)}$, represents the model?¡¥s prediction of the *k*-entanglement measure corresponding to the quantum state ρ. An illustration of the MLP architecture is shown in Figure 1.

The training objective of the MLP is to minimize the mean squared error (MSE) between the predicted and true entanglement values, which can be expressed as

(10) $$L_{\mathrm{MLP}}=\frac{1}{N}\sum_{i=1}^{N}{\left({\hat{E}}_{i}-E_{i}\right)}^{2},$$

where *N* denotes the number of training samples, and ${\hat{E}}_{i}$ and $E_{i}$ represent the predicted and true entanglement values of the *i*-th sample, respectively. The MLP thus learns the functional mapping from the density matrix ρ to its corresponding *k*-entanglement measure $E_{w}^{\left(k,n\right)}\left(\rho \right)$. During training, the network weights $W^{\left(l\right)}$ and biases $b^{\left(l\right)}$ are iteratively updated, enabling the model to progressively approximate the target function by the universal function approximation capability of neural networks. Theoretically, an MLP with at least one hidden layer and sufficient neurons, or a deep multilayer MLP, can approximate any continuous function to arbitrary precision, effectively learning the high-dimensional nonlinear mapping from density matrices to entanglement measures. MLP excels at modeling global nonlinear dependencies, integrating relationships across all elements of the density matrix. However, due to the absence of prior assumptions about local correlation patterns, they have limited ability to capture local dependencies between adjacent elements, which encode coherence or correlation information among subsystems in the quantum state. To overcome this limitation, CNN is introduced to complementarily model these local patterns.

**Figure 2:**
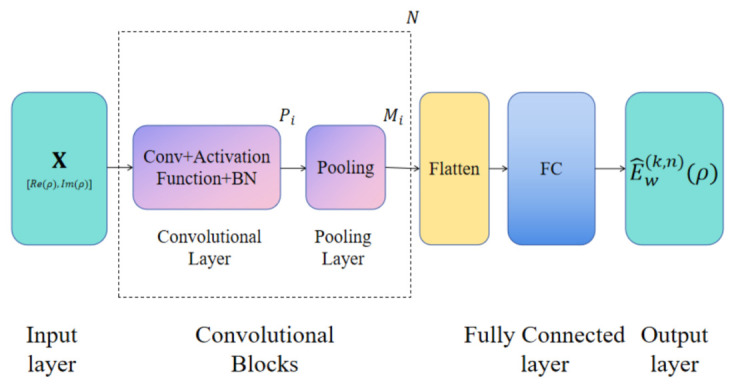
Illustration of the convolutional neural network (CNN) architecture. The input tensor X, consisting of two channels ℜ(ρ) and ℑ(ρ), is processed through *N* convolutional blocks (Conv-Activation function-BN-Pooling) to learn nonlinear features from the chosen matrix representation of the density operator. The flattened feature map is then passed through fully connected layers to produce the predicted *k*-entanglement measure ${\hat{E}}_{w}^{\left(k,n\right)}\left(\rho \right)$. The convolutional locality in this architecture should not be interpreted as physical locality in the multipartite quantum system.

**(b) CNN.** The CNN is a deep learning architecture widely used as a nonlinear function approximator for high-dimensional structured data. In this work, it is employed as a representation-learning model for matrix-valued inputs constructed from the density matrix. We emphasize that the density matrix is not regarded as an image with intrinsic physical pixel adjacency. Although a density matrix can be arranged as a two-dimensional array after choosing a computational basis, the neighboring relation between its matrix entries depends on the chosen basis and ordering convention. Therefore, the convolutional locality used by the CNN should be understood as an architectural inductive bias for the chosen matrix representation, rather than as physical locality in the multipartite quantum system. CNNs learn hierarchical representations of input data through convolutional operations and nonlinear transformations. Their core mechanism is based on local connectivity and weight sharing, which provide an efficient parameterization of high-dimensional nonlinear mappings. A local receptive field means that each neuron in a convolutional layer is connected only to a small subset of the input variables under the chosen matrix ordering. As multiple convolutional layers are stacked, the effective receptive field increases, enabling the model to construct increasingly abstract features of the chosen input representation. The overall CNN architecture typically consists of multiple convolutional blocks stacked sequentially. Each block comprises a convolutional layer, a nonlinear activation function, a batch normalization (BN) layer, and optionally a pooling layer. The intra-block processing pipeline can be summarized as follows:

(11) $${\left[\mathrm{Conv}\to \mathrm{Activation}\;\mathrm{function}\to \mathrm{BN}\right]}^{P_{i}}\to {\left[\mathrm{Pooling}\right]}^{M_{i}},$$

where $P_{i}$ denotes the number of consecutive convolutional layers and $M_{i}$ denotes the number of pooling layers in the *i*-th block. After passing through *N* convolutional blocks, the feature maps are flattened and fed into fully connected layers and a final regression output layer to generate the predicted entanglement measure. The overall architecture of the CNN model employed in this work is illustrated in Figure 2.

In general, the input to a CNN is a three-dimensional tensor, $X=\left[X_{i,j,c}\right]\in R^{H\times W\times C},$ where *H* and *W* denote the height and width of the input, and *C* is the number of channels. In this work, the input tensor is constructed from the real and imaginary parts of the quantum state density matrix ρ: $X=\left[\;ℜ\left(\rho \right),\;ℑ\left(\rho \right)\;\right]\in R^{2^{n}\times 2^{n}\times 2},$ with the first channel corresponding to ℜ(ρ) and the second to ℑ(ρ). This representation preserves the real and imaginary components of the complex density matrix in a form suitable for real-valued neural network input, while its spatial adjacency is determined by the chosen computational-basis ordering. Let $H^{\left(l-1\right)}$ and $H^{\left(l\right)}$ denote the input and output feature maps of the *l*-th convolutional layer, respectively: $H^{\left(0\right)}=X,\;H^{\left(l\right)}=\left[H_{i,j,k}^{\left(l\right)}\right]\in R^{H_{l}\times W_{l}\times C_{l}},$ where $H_{l}$, $W_{l}$, and $C_{l}$ denote the height, width, and number of channels of the output feature map. The forward propagation of a convolutional layer is given by

(12) $$H_{i,j,k}^{\left(l\right)}=f\;\left(\sum_{c=1}^{C_{l-1}}\sum_{p=1}^{K_{h}^{\left(l\right)}}\sum_{q=1}^{K_{w}^{\left(l\right)}}W_{p,q,c,k}^{\left(l\right)}\;H_{i+p-1,\;j+q-1,\;c}^{\left(l-1\right)}+b_{k}^{\left(l\right)}\right),$$

where $W_{p,q,c,k}^{\left(l\right)}$ and $b_{k}^{\left(l\right)}$ are the convolutional kernel weights and biases, $K_{h}^{\left(l\right)}$ and $K_{w}^{\left(l\right)}$ are the kernel height and width, and f(·) is the ReLU activation function f(x)=max(0,x). This process slides local kernels over the input feature maps, performing weighted aggregation and nonlinear mapping, effectively capturing local coupling patterns among adjacent elements in the density matrix and thus extracting local entanglement features of the quantum state. The feature maps are then normalized using batch normalization to improve numerical stability and accelerate convergence:

(13) $${\tilde{H}}_{i,j,k}^{\left(l\right)}=\gamma_{k}^{\left(l\right)}\frac{H_{i,j,k}^{\left(l\right)}-\mu_{k}^{\left(l\right)}}{\sqrt{{\left(\sigma_{k}^{\left(l\right)}\right)}^{2}+\epsilon }}+\beta_{k}^{\left(l\right)},$$

where $\mu_{k}^{\left(l\right)}$ and $\sigma_{k}^{\left(l\right)}$ are the mean and standard deviation of the *k*-th channel within the current batch, $\gamma_{k}^{\left(l\right)}$ and $\beta_{k}^{\left(l\right)}$ are learnable parameters, and ϵ is a small constant to avoid division by zero. Spatial downsampling is then performed via max pooling, $H_{i,j,k}^{\left(l+1\right)}=\max_{\left(p,q)\in R(i,j\right)}{\tilde{H}}_{p,q,k}^{\left(l\right)},$ where R(i,j) denotes an r×r neighborhood centered at (i,j). Pooling reduces the dimension of feature maps and improves the numerical efficiency of the subsequent fully connected layers. After several convolutional blocks, the high-level feature maps are flattened and fed into a fully connected layer, $z^{\left(L\right)}=\phi \;\left(W^{\left(L\right)}\mathrm{vec}\left(H^{\left(L-1\right)}\right)+b^{\left(L\right)}\right),$ where ϕ(·) is the ReLU activation, and $W^{\left(L\right)}$ and $b^{\left(L\right)}$ are the weights and biases of the fully connected layer. The final output layer produces the predicted *k*-entanglement measure through a linear mapping: ${\hat{E}}_{w}^{\left(k,n\right)}\left(\rho \right)=w_{\mathrm{out}}^{⊤}z^{\left(L\right)}+b_{\mathrm{out}}.$ During training, the model minimizes the MSE,

(14) $$L_{\mathrm{CNN}}=\frac{1}{N}\sum_{i=1}^{N}{\left({\hat{E}}_{w,i}^{\left(k,n\right)}-E_{w,i}^{\left(k,n\right)}\right)}^{2},$$

using the Adam optimizer, along with dropout and early stopping on a validation set to prevent overfitting.

The CNN model is therefore used as a data-driven nonlinear approximation method for the mapping $\rho \mapsto E_{w}^{\left(k,n\right)}\left(\rho \right)$. Its convolutional structure provides parameter sharing and hierarchical feature extraction for the chosen matrix representation of ρ, but it does not by itself encode physical locality or subsystem permutation symmetry. We also note that the standard CNN architecture used here does not explicitly enforce subsystem permutation invariance. For a permutation π of equivalent subsystems, the transformed state can be written as $\rho_{\pi }=U_{\pi }\rho U_{\pi }^{†}$, where $U_{\pi }$ is the corresponding subsystem permutation unitary. The value of the *k*-entanglement measure is invariant under such relabeling when the associated partition structure is transformed consistently. However, this invariance is not guaranteed by a standard CNN, MLP, or LightGBM model unless it is imposed by architecture design, data augmentation, or an explicit symmetry constraint. Therefore, permutation robustness should be tested separately. Symmetry-aware models, such as DeepSets, graph neural networks, permutation-equivariant neural networks, or transformer architectures with suitable symmetry constraints, may provide more natural ways to incorporate this property in future work.

**(c) LightGBM.** Unlike neural network models such as MLP and CNN, gradient boosting methods combine multiple weak learners in an additive manner to approximate nonlinear targets, often achieving strong performance on structured tabular data while maintaining relatively good interpretability. To evaluate the effectiveness of non-neural network approaches for predicting quantum entanglement measures, we adopt LightGBM, a decision-tree-based gradient boosting framework. LightGBM constructs a sequence of regression trees iteratively, where each tree refines the current prediction using gradient-based optimization of the loss function.

Let the training dataset be ${\left\{\left(h_{i},E_{i}\right)\right\}}_{i=1}^{N}$, where $h_{i}\in R^{d}$ denotes the feature vector of the *i*-th sample, obtained from the flattened density matrix or extracted features, and $E_{i}=E_{w}^{\left(k,n\right)}\left(\rho_{i}\right)$ represents the target entanglement measure. The training objective is to minimize the mean squared error (MSE)

(15) $$L_{\mathrm{LGBM}}=\frac{1}{N}\sum_{i=1}^{N}{\left({\hat{E}}_{i}-E_{i}\right)}^{2},$$

where ${\hat{E}}_{i}$ denotes the predicted value for the *i*-th sample. Atboosting iteration *t*, the prediction is updated as

(16) $${\hat{E}}_{i}^{\left(t\right)}={\hat{E}}_{i}^{\left(t-1\right)}+\eta \;f_{t}\left(h_{i}\right),$$

where η is the learning rate and $f_{t}$ is the *t*-th regression tree. To construct the tree, LightGBM performs a second-order Taylor expansion of the loss function around the current prediction. Ignoring constant scaling factors that do not affect the tree optimization, the first- and second-order derivatives of the MSE loss can be written as

(17) $$g_{i}=\frac{\partial L}{\partial {\hat{E}}_{i}^{\left(t-1\right)}}\propto {\hat{E}}_{i}^{\left(t-1\right)}-E_{i},\;h_{i}=\frac{\partial^{2}L}{\partial {\left({\hat{E}}_{i}^{\left(t-1\right)}\right)}^{2}}=1.$$

Using these derivatives, the approximate objective for the *t*-th tree becomes

(18) $$L^{\left(t\right)}\approx \sum_{i=1}^{N}\left[g_{i}f_{t}\left(h_{i}\right)+\frac{1}{2}h_{i}f_{t}^{2}\left(h_{i}\right)\right]+\Omega \left(f_{t}\right),$$

where $\Omega (f_{t})$ is a regularization term penalizing overly complex trees. During tree construction, LightGBM evaluates candidate splits using the gain function. For a parent node split into left child *L* and right child *R*, the gain is

(19) $$\mathrm{Gain}=\frac{1}{2}\left(\frac{G_{L}^{2}}{H_{L}+\lambda }+\frac{G_{R}^{2}}{H_{R}+\lambda }-\frac{{\left(G_{L}+G_{R}\right)}^{2}}{H_{L}+H_{R}+\lambda }\right)-\gamma ,$$

where $G_{L}=\sum_{i\in L}g_{i}$, $G_{R}=\sum_{i\in R}g_{i}$ and $H_{L}=\sum_{i\in L}h_{i}$, $H_{R}=\sum_{i\in R}h_{i}$. Here λ is the $L_{2}$ regularization coefficient and γ is the split penalty. After determining the tree structure, the optimal output weight of leaf node *j* is

(20) $$w_{j}^{*}=-\frac{G_{j}}{H_{j}+\lambda },$$

where $G_{j}=\sum_{i\in j}g_{i}$ and $H_{j}=\sum_{i\in j}h_{i}$ denote the aggregated gradients of samples in leaf *j*. For an input feature vector h, the tree output is

(21) $$f_{t}\left(h\right)=w_{j(h)}^{*}.$$

Finally, the LightGBM prediction is obtained by summing the outputs of all boosting trees

(22) $${\hat{E}}_{w}^{\left(k,n\right)}\left(\rho \right)=\sum_{t=1}^{T}\eta \;f_{t}\left(h\right),$$

where *T* denotes the number of boosting iterations. Compared with traditional gradient boosting implementations, LightGBM employs histogram-based feature binning and a leaf-wise tree growth strategy, which significantly improves computational efficiency and scalability for high-dimensional datasets. The entire LightGBM training procedure is summarized in Algorithm 1.

LightGBM adopts a leaf-wise tree growth strategy together with a histogram-based approximate splitting algorithm, which enables both high predictive accuracy and efficient training. Continuous feature values are first discretized into a fixed number of bins, and gradient statistics within each bin are aggregated so that candidate splits are evaluated only at bin boundaries. This significantly reduces the number of candidate split points and accelerates tree construction. Unlike neural network models such as MLP or CNN, LightGBM does not rely on differentiable parametric mappings or local connectivity structures. Instead, it models nonlinear relationships by partitioning the feature space through an ensemble of decision trees. For quantum entanglement prediction, this framework allows us to analyze feature splits and information gain to identify influential components of the density matrix, thereby providing interpretable insights into the structural features that contribute most to the predicted entanglement measure.
**Algorithm 1** LightGBM Training for *K*-Entanglement Prediction**Input:** Training dataset $D={\left\{\left(h_{i},E_{i}\right)\right\}}_{i=1}^{N}$, learning rate η, number of trees *T*, regularization parameters λ,γ**Output:** Trained additive tree model ${\hat{E}}_{w}^{\left(k,n\right)}\left(\rho \right)$ 1:**Initialize predictions:** ${\hat{E}}_{i}^{\left(0\right)}\leftarrow 0$ for i=1,…,N 2:**for** t=1 to *T* **do** 3:    **Step 1: Compute gradients and Hessians** 4:    **for** i=1 to *N* **do** 5:       Ignoring constant scaling factors of the MSE loss: 6:       $g_{i}\leftarrow {\hat{E}}_{i}^{\left(t-1\right)}-E_{i}$ 7:       $h_{i}\leftarrow 1$ 8:    **end for** 9:    **Step 2: Construct regression tree $f_{t}$**  10:    Discretize each feature into histogram bins  11:    Initialize root node containing all samples  12:    **while** there exist splittable leaf nodes **do**  13:       **for** each leaf node **do**  14:           **for** each feature *j* **do**  15:              **for** each candidate split *s* **do**  16:                 Compute gradient sums:  17:                 $G_{L}=\sum_{i\in L}g_{i}$, $H_{L}=\sum_{i\in L}h_{i}$  18:                 $G_{R}=\sum_{i\in R}g_{i}$, $H_{R}=\sum_{i\in R}h_{i}$  19:                 Compute split gain:  20:                  $\mathrm{Gain}=\frac{1}{2}\;\left(\frac{G_{L}^{2}}{H_{L}+\lambda }+\frac{G_{R}^{2}}{H_{R}+\lambda }-\frac{{\left(G_{L}+G_{R}\right)}^{2}}{H_{L}+H_{R}+\lambda }\right)-\gamma$  21:             **end for**  22:          **end for**  23:       **end for**  24:       Split the leaf node with the largest Gain  25:    **end while**  26:    **Step 3: Compute optimal leaf weights**  27:    **for** each leaf *j* **do**  28:       $w_{j}^{*}\leftarrow -\frac{G_{j}}{H_{j}+\lambda }$  29:    **end for**  30:    Assign tree output  31:    **for** i=1 to *N* **do**  32:       $f_{t}\left(h_{i}\right)\leftarrow w_{j(h_{i})}^{*}$  33:    **end for**  34:    **Step 4: Update predictions**  35:    **for** i=1 to *N* **do**  36:       ${\hat{E}}_{i}^{\left(t\right)}\leftarrow {\hat{E}}_{i}^{\left(t-1\right)}+\eta f_{t}\left(h_{i}\right)$  37:    **end for**  38:**end for**  39:**Return prediction model**  40:${\hat{E}}_{w}^{\left(k,n\right)}\left(\rho \right)=\sum_{t=1}^{T}\eta f_{t}\left(h\right)$

### 3.2. Stacking Ensemble Framework

**Figure 3:**
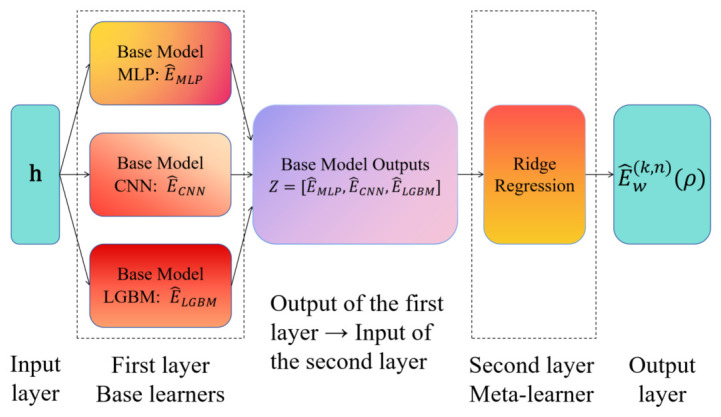
Illustration of the stacking ensemble framework used in this work. The first layer consists of three base models (MLP, CNN, and LightGBM), each independently predicting the quantum *k*-entanglement measure. Their predicted values are combined into a new feature vector, which serves as the input to the second-layer meta-learner (Ridge Regression) for final estimation. The arrow indicates that the outputs of the first-layer base models collectively form the input to the meta-learner in the second layer.

The previously introduced MLP, CNN, and LightGBM models capture the mapping from a quantum state density matrix to its *k*-entanglement measure from complementary perspectives. The MLP models global nonlinear dependencies, representing the overall structure of the quantum state. The CNN extracts local features from the density matrix, identifying entanglement patterns among subsystems. LightGBM uses a tree-based splitting mechanism to assess the contributions of individual matrix elements from a statistical standpoint. Each model provides unique information, and combining them can improve both predictive accuracy and robustness. To achieve this, we employ a stacking ensemble that integrates the three models into a unified framework (Figure 3).

Stacking consists of two layers. The first layer contains the base models, each independently producing predictions from the input features. The second layer is a meta-learner that aggregates these predictions and learns their optimal weighting. Let the set of base models be $B=\{f_{\mathrm{MLP}},\;f_{\mathrm{CNN}},\;f_{\mathrm{LGBM}}\},$ where each base model $f_{b}\left(\cdot \right)$ receives an input feature vector h (flattened or feature-extracted from the density matrix ρ) and outputs a predicted entanglement value:

(23) $${\hat{E}}_{b}=f_{b}\left(h\right),\;b\in B.$$

The predictions of the three base models are combined into a new feature vector, $Z={\left[{\hat{E}}_{\mathrm{MLP}},\;{\hat{E}}_{\mathrm{CNN}},\;{\hat{E}}_{\mathrm{LGBM}}\right]}^{⊤}\in R^{3},$ which serves as input to the second-layer meta-learner, generating the final prediction:

(24) $${\hat{E}}_{\mathrm{stack}}=f_{\mathrm{meta}}\left(Z\right).$$

We employ Ridge Regression as the meta-learner, whose model takes the form

(25) $$f_{\mathrm{meta}}\left(Z\right)=\sum_{b\in B}\alpha_{b}{\hat{E}}_{b}+\beta ,$$

where $\alpha_{b}$ are the fusion weights of the base models and β is the bias term. Ridge Regression introduces an $L_{2}$ regularization term in the loss function to constrain excessively large weights, preventing overfitting during model combination and mitigating instability caused by correlated base model predictions. The optimization objective is thus

(26) $$L_{\mathrm{meta}}=\frac{1}{N}\sum_{i=1}^{N}{\left({\hat{E}}_{\mathrm{stack},i}-E_{i}\right)}^{2}+\lambda \sum_{b\in B}\alpha_{b}^{2},$$

where λ is the regularization coefficient controlling the trade-off between model complexity and generalization performance.

For a fixed *n*-partite system and a fixed admissible value of *k* with 2≤k≤n, the output of the neural network is a scalar value ${\hat{E}}_{w}^{\left(k,n\right)}\left(\rho \right)$. In the present implementation, different admissible values k=2,…,n are handled by training separate models. Thus, the model is trained as a fixed-*k* regression model for the mapping $\rho \mapsto E_{w}^{\left(k,n\right)}\left(\rho \right)$, rather than as a multi-output model covering all *k* values simultaneously. This separate training strategy is physically motivated by the fact that different values of *k* correspond to different reference sets in the multipartite separability hierarchy and therefore to different entanglement criteria. In particular, k=2 is the relevant level for detecting and quantifying genuine multipartite entanglement, since 2-nonseparability is equivalent to genuine multipartite entanglement. At the other end, k=n corresponds to full separability, and *n*-nonseparability means that the state is entangled in the usual sense of being not fully separable. Intermediate values 2<k<n describe entanglement relative to intermediate levels of multipartite separability. Training an independent model for each fixed admissible *k* keeps the regression target physically and mathematically well defined, and allows one to deploy only the model corresponding to the desired entanglement level. A multi-output formulation for predicting several *k*-entanglement measures at once is possible in principle. For a fixed *n*, this would amount to predicting the vector $\left(E_{w}^{\left(2,n\right)}\left(\rho \right),E_{w}^{\left(3,n\right)}\left(\rho \right),\ldots ,E_{w}^{\left(n,n\right)}\left(\rho \right)\right)$. However, such a formulation would require labels for all admissible *k* values and a suitable multi-task loss or target normalization to balance the different numerical scales. Taking into account training costs and the fact that in practical applications we often do not need to know the values of all k-entanglement measures, this work does not include an implementation with multi-output formulations.

To prevent information leakage during training, the stacking framework uses a stratified *K*-fold cross-validation strategy with K=5. Each base model is trained on K−1 folds and generates out-of-fold (OOF) predictions on the remaining fold. Because each sample’s OOF prediction comes from a model that has not seen it during training, predictive independence is ensured. The OOF outputs from all folds are combined to form the training input for the meta-learner, ensuring that the meta-model is trained independently of the final test set. After this procedure, each base model is refitted on the full training dataset, and the meta-learner uses the corresponding OOF outputs to compute the final weighted prediction. From a physical perspective, the stacking ensemble can be interpreted as a collaborative approach for modeling multi-scale entanglement features. By integrating and weighting predictions across different feature spaces, the framework captures both global and local correlations in quantum states. This enables efficient and accurate prediction of *k*-entanglement measures at low computational cost. The ensemble enhances generalization and stability while providing a scalable machine learning strategy for rapid characterization of entanglement in multipartite quantum systems.

### 3.3. Dataset Construction and Evaluation Framework

To evaluate machine learning models for predicting the *k*-entanglement of multipartite quantum systems, this work introduces a general framework for dataset generation and model assessment. The framework handles quantum states with any number of subsystems and finite local dimensions of at least two. Standardized datasets generated by the framework enable training, testing, and benchmarking of models across diverse system configurations.

Given a system structure $\delta =(d_{1},d_{2},\ldots ,d_{n})$, the corresponding Hilbert space is

$$H=H_{1}⊗H_{2}⊗\cdots ⊗H_{n},\;\mathrm{with}\;\dim H_{j}=d_{j}\;\mathrm{and}\;\dim\left(H\right)=D=\prod_{j=1}^{n}d_{j}.$$

The witness-based quantity $E_{w}^{\left(k,n\right)}$ is defined for arbitrary density operators on H. Therefore, in the main benchmark, we generate general mixed states $\rho \in C^{D\times D}$ satisfying

$$\rho =\rho^{†},\;\rho \geq 0,\;\mathrm{Tr}\left(\rho \right)=1,$$

and compute their corresponding witness-based *k*-entanglement labels by the method proposed in [21], for each fixed admissible value of *k* with 2≤k≤n. This gives supervised learning samples of the form $\left(\rho ,{\tilde{E}}_{w}^{\left(k,n\right)}\left(\rho \right)\right),$ where ${\tilde{E}}_{w}^{\left(k,n\right)}\left(\rho \right)$ denotes the computationally realized value obtained from the finite witness database. In practice, we first generate a random complex matrix

$$A=\left(A_{ij}\right)\in C^{D\times D},\;A_{ij}=a_{ij}^{\left(r\right)}+i\;a_{ij}^{\left(i\right)},\;a_{ij}^{\left(r\right)},a_{ij}^{\left(i\right)}∼U\left(0,1\right),$$

and then construct a density matrix by $\rho =\frac{A^{†}A}{\mathrm{Tr}(A^{†}A)}.$ This construction automatically guarantees positivity and unit trace, and provides a generic benchmark distribution of valid mixed quantum states. Since $E_{w}^{\left(k,n\right)}$ is defined on the full density-operator space, the purpose of this dataset is to test the surrogate-learning framework on a broad class of density matrices, rather than to restrict the model to a particular experimentally motivated state family. This choice helps reduce over-specialization to a narrow state distribution. For example, training only on noisy GHZ states, thermal states, tensor-network states, or circuit states from a fixed hardware architecture may improve performance on that specific distribution but may also reduce generality for broader density-matrix inputs.

For feature construction, in order for machine learning models to directly process the data, complex-valued density matrices need to be converted into real-valued feature representations. As the total Hilbert space dimension is $D=\prod_{j=1}^{n}d_{j}$, for each density matrix $\rho \in C^{D\times D}$, the elements of the upper-triangular part (including the diagonal) can be separated into real and imaginary components and flattened row-wise into a real-valued vector. The diagonal elements are nonnegative with sum 1, contributing D−1 features; the off-diagonal elements in the upper-triangle number $\left(D-1\right)+\left(D-2\right)+\cdots +1=\frac{D(D-1)}{2}$ complex entries, each contributing two real features (real and imaginary parts). Therefore, the total number of real features is $2\times \frac{D(D-1)}{2}+D-1=D^{2}-1$, which matches the real dimension of Hermitian matrices with unit trace. To accommodate different model architectures, two feature representations are adopted. For models that accept vector inputs, such as MLP and LightGBM, the flattened real vector $h\in R^{D^{2}-1}$ is used directly. For CNN models, the real and imaginary parts of the density matrix are treated as two separate input channels $\left[ℜ\left(\rho \right),ℑ\left(\rho \right)\right]\in R^{D\times D\times 2}$, allowing convolutional operations to exploit the spatial locality of the matrix for feature extraction. Both representations preserve the physical constraints while leveraging the structural learning advantages of different architectures. The dataset is split into training and test sets with a fixed ratio, where 80% of the data is used for training and 20% for testing. The entanglement distribution is maintained consistently during the split to ensure independent and statistically reliable model evaluation.

Before introducing the evaluation metrics, we clarify the meaning of the reference labels used in the regression task. The labels used for supervised learning are the finite-database witness-based quantities ${\tilde{E}}_{w}^{\left(k,n\right)}$, rather than the abstract exact functional $E_{w}^{\left(k,n\right)}$ itself. Accordingly, the trained model should be interpreted as approximating the numerical estimator ${\tilde{E}}_{w}^{\left(k,n\right)}$. The finite witness database approximation error has been discussed in [21], where it is reported to be approximately of order $10^{-4}$. The present work focuses on the regression accuracy of the machine learning prediction ${\hat{E}}^{\left(k,n\right)}$ with respect to ${\tilde{E}}_{w}^{\left(k,n\right)}$.

To evaluate this regression performance, we adopt three standard metrics: mean squared error (MSE), mean absolute error (MAE), and the coefficient of determination ($R^{2}$):

(27) $$\mathrm{MSE}=\frac{1}{N}\sum_{i=1}^{N}{\left({\hat{E}}_{i}-E_{i}\right)}^{2},\;\mathrm{MAE}=\frac{1}{N}\sum_{i=1}^{N}\left|{\hat{E}}_{i}-E_{i}\right|,\;R^{2}=1-\frac{\sum_{i=1}^{N}{\left({\hat{E}}_{i}-E_{i}\right)}^{2}}{\sum_{i=1}^{N}{\left(E_{i}-\bar{E}\right)}^{2}}.$$

Here, $E_{i}={\tilde{E}}_{w}^{\left(k,n\right)}\left(\rho_{i}\right)$ denotes the finite-database witness-based reference label, ${\hat{E}}_{i}={\hat{E}}^{\left(k,n\right)}\left(\rho_{i}\right)$ denotes the corresponding machine learning prediction, and $\bar{E}$ is the mean of the reference labels. The MSE measures the average squared deviation between the predicted values and the finite-database reference labels, and therefore penalizes large errors more strongly. The MAE measures the average absolute deviation and gives a direct measure of the typical prediction error. The coefficient of determination $R^{2}$ measures the proportion of variance in the reference labels explained by the model. These three metrics provide complementary views of regression accuracy.

In addition to prediction accuracy, the average inference time on the test set, denoted as time, is reported to quantify the computational cost during the prediction phase. This metric reflects the efficiency of the trained model at deployment and complements the standard accuracy measures. While MSE, MAE, and $R^{2}$ evaluate approximation quality from different perspectives, time characterizes computational overhead during inference.

By jointly considering MSE, MAE, $R^{2}$, and time, the models can be compared in terms of both predictive performance and computational efficiency. Such a multi-criteria evaluation provides a systematic basis for model comparison and subsequent application to multipartite quantum systems.

## 4. Numerical Results

To verify the effectiveness of the proposed dataset construction procedure and evaluation framework, and to systematically analyze the predictive performance of machine learning models under different system sizes and entanglement orders, we conduct comprehensive experiments on nine representative multipartite quantum systems of qubit number ≤4. Each system is characterized by a structural parameter $\delta_{k}$, which defines an independent supervised learning task. The goal is to learn the functional mapping from a density matrix ρ to its corresponding *k*-entanglement measure $E_{w,m}^{\left(k,n\right)}\left(\rho \right)=E_{w}^{\left(k,n\right)}\left(\rho \right)$; here *m* indicates the qubit number. The systems $\delta_{k}$ considered in the experiments are ${\left(2,2,2\right)}_{k=2},$ ${\left(2,2,2\right)}_{k=3},$ ${\left(2,4\right)}_{k=2},$ for m=3 and ${\left(2,2,2,2\right)}_{k=2},$ ${\left(2,2,2,2\right)}_{k=3},$ ${\left(2,2,2,2\right)}_{k=4},$ ${\left(2,4,2\right)}_{k=2},$ ${\left(2,4,2\right)}_{k=3},\;{\left(2,8\right)}_{k=2}$ for m=4. The three-qubit tripartite and four-qubit four-partite systems correspond to (2,2,2) and (2,2,2,2) respectively. In contrast, structures such as (2,4), (2,8) and (2,4,2) correspond to the three-qubit bipartite, four-qubit bipartite system and four-qubit tripartite system, respectively. For example, the Hilbert space of system (2,4,2) can be written as $C^{2}⊗\left(C^{2}⊗C^{2}\right)⊗C^{2}$, in which the local dimension four arises from combining two qubit subsystems. This design ensures physical consistency and simultaneously provides diverse scenarios for studying how local dimensions influence entanglement complexity and learning difficulty.

Following the quantum-state sampling procedure described earlier, we generate randomly mixed-state samples that satisfy Hermiticity, unit trace, and positive semidefinite constraints. This process yields the dataset $D_{\delta_{k}}={\left\{\left(\rho_{i},E_{w,m}^{\left(k,n\right)}\left(\rho_{i}\right)\right)\right\}}_{i=1}^{N},N=30,000,$ with an 80%-20% split between the training and test sets. During sampling, we maintain the entanglement-degree distribution to ensure statistical comparability across systems. The main computational bottleneck generally appears not in the generation of quantum states but in the exact evaluation of the *k*-entanglement measure for each generated sample state. But for our situation, this difficulty is overcome by employing the software of computing highly accurate approximation ${\tilde{E}}_{w,m}^{\left(k,n\right)}$ of $E_{w,m}^{\left(k,n\right)}$ proposed in [21], which uses a preconstructed database $\tilde{\mathrm{EW}}\left(H\right)$ as in Equation (7), with $H=C^{2}⊗C^{2}⊗C^{2}$ and $C^{2}⊗C^{2}⊗C^{2}⊗C^{2}$. Our sample database is available at [58], with states stored in rho_data.npz and the corresponding labels in results.csv.

Two types of feature representations are used to encode the quantum states. The first representation flattens the diagonal entries of the density matrix and the real and imaginary parts of its upper-triangular off-diagonal entries into a one-dimensional vector h, which serves as the input for the MLP and LightGBM models. The second representation retains the two-dimensional structure of the density matrix and treats its real and imaginary parts as two separate channels for the CNN. This design enables the CNN to extract local spatial patterns and spectral correlations. Together, these representations match the architectural strengths of the respective models while preserving all physical constraints.

After constructing the dataset, all models are trained and evaluated on a unified hardware platform to ensure a fair comparison. Experiments are conducted on a workstation equipped with an Intel Xeon Gold processor, 32 GB RAM, and an NVIDIA RTX 3090 GPU running Windows 10. The models are implemented in Python 3.12.7 using PyTorch 2.9.1, scikit-learn 1.8.0, and LightGBM 4.6.0. Deep neural network models are trained with GPU acceleration, while tree-based and classical machine learning models are executed on the CPU.

The model architectures and hyperparameter choices were described previously. For completeness, we summarize the key settings used in the experiments. The MLP contains three fully connected layers with hidden dimensions 256, 128, and 64. It uses ReLU activations, batch normalization, and dropout with a rate of 0.2. Weights are initialized using the Kaiming scheme and biases are set to zero. The CNN consists of three convolutional layers with 16, 32, and 64 channels and 3×3 kernels. These layers are followed by activation, batch normalization, max pooling, and dropout with a rate of 0.2. The flattened features are then passed through two fully connected layers with 256 and 64 units and dropout rates of 0.25 and 0.1. Both deep models are trained using the Adam optimizer with an initial learning rate of $10^{-3}$, together with learning-rate scheduling and early stopping to improve convergence stability. The LightGBM model uses gradient-boosted decision trees with a learning rate of 0.007, a maximum of 32 leaves, a minimum of four samples per leaf, a feature-sampling ratio of 0.9, a data-sampling ratio of 0.9, a sampling frequency of three, and an $L_{2}$ regularization strength of 0.5. This configuration enables efficient nonlinear decision boundaries in high-dimensional feature spaces. After training the base models, we construct a stacked ensemble to combine their complementary inductive biases. The meta-learner is a Ridge Regression model that takes the validation-set predictions of the MLP, CNN, and LightGBM as input. The regularization strength is selected by a logarithmic search over the interval from $10^{-5}$ to $10^{4}$. Five-fold cross-validation is performed within a standardized pipeline to achieve a balance between noise suppression and model flexibility. All models are trained using the MSE objective following the supervised learning procedure outlined earlier.

We first perform a comprehensive evaluation of all models on an independent test set using four metrics: MAE, MSE, $R^{2}$, and time. These metrics jointly assess prediction accuracy, trend-capturing capability, and computational efficiency in the task of *k*-entanglement prediction. The detailed results are reported in Table 1, Table 2, Table 3 and Table 4.

The reported results indicate that all models achieve relatively high predictive accuracy on the multipartite *k*-entanglement estimation task. Typically, the MAE is at the $10^{-3}$ level, the MSE is around $10^{-5}$, and the $R^{2}$ scores fall within [0.875,1]. Here, MAE reports the average absolute difference between predictions and ground-truth values, so an MAE at the $10^{-3}$ level indicates a small typical pointwise error. By contrast, MSE squares the residuals and therefore penalizes larger errors much more strongly; an MSE around $10^{-5}$ suggests that noticeably large errors are not frequent enough to dominate the squared-error metric. Table 1 also indicates that prediction difficulty varies across system settings. For the ${\left(2,4\right)}_{k=2}$ dataset, both the MLP and CNN reach MAE at the $10^{-4}$ level, and the ensemble model achieves a similar MAE. Equivalently, on this dataset the average absolute difference between predicted and exact values is about $10^{-4}$, which is lower than the MAE observed on the other datasets. Regarding squared-error behavior, Table 2 shows that the ensemble model achieves MSE in the $10^{-6}$ range across the datasets reported. Since MSE grows rapidly when a model produces a small number of noticeably wrong predictions, MSE values consistently in the $10^{-6}$ range suggest that large-error cases are relatively rare for the ensemble, and its squared-error level remains low across different datasets. In addition, trend-capturing performance is reflected by $R^{2}$. Table 3 shows that the $R^{2}$ values of the MLP and CNN models are almost uniformly close to one, indicating a strong ability to learn the complex nonlinear correlation structure inherent in multipartite quantum states. LightGBM yields slightly lower $R^{2}$ scores but still maintains high overall accuracy; the remaining gap may be related to the inductive bias of tree-based models when approximating smooth high-order correlations.

More importantly, the aspect of computational efficiency, which is a key focus of this work, is clearly reflected in Table 4. All models achieve inference times in the range of $10^{-4}$–$10^{-5}$ seconds, already reaching or close to a real-time computational regime. Compared with existent multipartite *k*-entanglement measure computation methods that typically require minutes to days, this level of acceleration is transformative. LightGBM achieves the fastest inference due to its lightweight tree structure, while the ensemble model incurs a slightly higher overhead due to aggregation across multiple predictors. Nevertheless, its inference time remains below $10^{-4}$ seconds, still meeting the requirements of rapid *k*-entanglement estimation. These results show that high prediction accuracy and high computational efficiency can indeed be achieved simultaneously, giving the method direct applicability in large-scale quantum multipartite analysis and real-time experimental data processing. Taken together, the four metrics reveal a clear performance landscape: the MLP and CNN models deliver the highest predictive accuracy, LightGBM holds a distinct advantage in computational speed, and the ensemble model achieves the best balance between accuracy, stability, and efficiency. With its MSE stably reaching $10^{-6}$ and its inference time remaining in the sub-millisecond range, the ensemble model provides an excellent “accuracy–efficiency” trade-off and thus serves as an ideal predictor for *k*-entanglement in subsequent physical applications.

The Werner states are useful and important states in quantum information theory, in which the *k*-entanglement can be identified exactly. Thus, one usually utilizes Werner states to test the ability of entanglement criteria. To test the generalization performance of our ML models, we evaluate further our trained ensemble model to the *k*-entanglement measures of three-qubit and four-qubit Werner states, and compare them with the corresponding true value of ${\tilde{E}}_{w,m}^{\left(k,n\right)}$.

**Figure 4:**
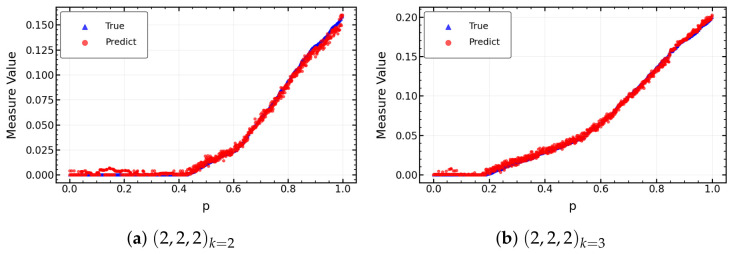
Comparison between the true values ${\tilde{E}}_{w,3}^{\left(k,3\right)}\left(\rho_{W_{3}}\left(p\right)\right)$ and predicted *k*-entanglement values on the 3-qubit Werner state test set. Blue triangles denote the true values, while red circles represent the predictions produced by the ensemble machine learning model.

**Figure 5:**
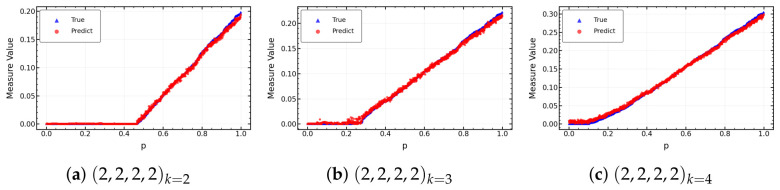
Comparison between the true values ${\tilde{E}}_{w,4}^{\left(k,4\right)}\left(\rho_{W_{4}}\left(p\right)\right)$ and the predicted values on the 4-qubit Werner state test set.

Recall that, for n>2, an *n*-qubit Werner state is defined as

$$\rho_{W_{n}}\left(p\right)=p\;|\phi \rangle \langle \phi |+\frac{1-p}{2^{n}}I,$$

where $|\phi \rangle =\frac{1}{\sqrt{2}}\left({|0\rangle }^{⊗n}+{|1\rangle }^{⊗n}\right),$ *I* denotes the $2^{n}\times 2^{n}$ identity matrix, and the parameter p∈[0,1] specifies the weight of the pure state |ϕ〉 within the mixed state. For the three-qubit tripartite scenario, $\rho_{W_{3}}\left(p\right)$ is known to be entangled (k=3) if and only if $p>\frac{1}{5}$ [59,60]. Ref. [61] showed that $\rho_{W_{3}}\left(p\right)$ is genuinely multipartite entangled, i.e., 2-entangled, if and only if $p>\frac{3}{7}$. For the four-qubit four-partite system, exact boundaries for *k*-entanglement have been established: $\rho_{W_{4}}\left(p\right)$ remains entangled if and only if $p>\frac{1}{9}$ [59,60], is 3-entangled if and only if $p>\frac{1}{5}$ [62], is genuinely entangled if and only if $p>\frac{7}{15}$ [61]. Correspondingly, to validate the model, five system configurations are considered: ${\left(2,2,2\right)}_{k=2}$, ${\left(2,2,2\right)}_{k=3}$, ${\left(2,2,2,2\right)}_{k=2}$, ${\left(2,2,2,2\right)}_{k=3}$, and ${\left(2,2,2,2\right)}_{k=4}$. Each configuration is represented by 1000 randomly generated Werner state samples, with their *k*-entanglement measures computed using rigorous numerical methods in [21]. Then the model predictions are compared pointwise with the true values, allowing a direct evaluation of how well the model captures the variation in entanglement structure. The comparisons are displayed in Figure 4 and Figure 5, where the horizontal axis corresponds to the Werner state parameter *p*, and average inference times across all configurations are summarized in Table 5.

As shown in Figure 4 and Figure 5, the proposed ensemble model accurately reproduces the reference *k*-entanglement behavior for both three-qubit and four-qubit Werner states. Over the whole parameter range, the predicted values agree well with the theoretical separability thresholds and the corresponding witness-based *k*-entanglement values. In particular, the model correctly identifies the zero-entanglement regions and gives positive predictions after the corresponding thresholds are crossed. For the three-qubit Werner states, in the ${\left(2,2,2\right)}_{k=2}$ case, the state is 2-separable for $0\leq p\leq \frac{1}{5}$, and hence the theoretical 2-entanglement measure is zero in this interval. The ensemble model gives values that are numerically close to zero in the same region. Similarly, in the ${\left(2,2,2\right)}_{k=3}$ case, the state is 3-separable for $0\leq p\leq \frac{3}{7}$, and the predicted 3-entanglement measure also remains close to zero within this separable interval. For the four-qubit Werner states, the model also captures the hierarchical transition of *k*-entanglement. In the ${\left(2,2,2,2\right)}_{k=2}$ case, $\rho_{W_{4}}\left(p\right)$ is 2-separable for $0\leq p\leq \frac{7}{15}$. The predicted 2-entanglement measure remains close to zero in this interval and becomes positive when $p>\frac{7}{15}$, corresponding to the onset of genuine multipartite entanglement. In the ${\left(2,2,2,2\right)}_{k=3}$ case, the model gives nearly zero values for $0\leq p\leq \frac{1}{5}$ and positive values after the 3-separability threshold is crossed. In the ${\left(2,2,2,2\right)}_{k=4}$ case, the Werner state is fully separable for $0\leq p\leq \frac{1}{9}$, and the predicted 4-entanglement measure becomes positive when $p>\frac{1}{9}$. These results show that the ensemble model can identify the separable regions and reproduce the transition from zero to nonzero *k*-entanglement in these symmetric benchmark families.

The computational advantage is also significant. As reported in Table 5, the inference time for each Werner state is at the millisecond level, whereas the direct evaluation using the software tool in [21] requires tens to hundreds of seconds for the tested three-qubit and four-qubit cases. This corresponds to a speed-up of more than five orders of magnitude. Therefore, after training, the learned surrogate model provides a fast numerical approximation to the witness-based *k*-entanglement measure. This speed-up is particularly useful for repeated evaluations, such as parameter scans, state-space exploration, and iterative numerical studies. Nevertheless, the predictions should be understood as surrogate numerical estimates within the tested distributions, rather than as independent rigorous entanglement certificates.

Since Werner states form highly symmetric one-parameter families, the corresponding tests are used here mainly as consistency checks for the known threshold behavior, rather than as a comprehensive validation over general multipartite state spaces. To further examine the predictive performance of the learned surrogate model beyond Werner states and randomly generated density matrices, we introduce an additional one-parameter family of four-qubit noisy circuit-generated states. This family is obtained from a fixed non-symmetric circuit-prepared pure state followed by local amplitude-damping noise. It therefore provides a more structured and physically motivated test distribution. This construction follows the standard modeling strategy of noisy parameterized quantum circuits: a finite-depth circuit with local rotation layers and entangling gates is used for state preparation, and a local amplitude-damping channel is then applied as a standard energy-relaxation noise model [2,63,64,65].

The construction consists of two steps. First, starting from the initial product state |0000〉, we apply a finite-depth random parameterized circuit $U_{\mathrm{ns}}$. More explicitly, $U_{\mathrm{ns}}$ is defined by

(28) $$U_{\mathrm{ns}}=\prod_{\ell =1}^{L}\left[C_{2\to 3}C_{1\to 2}C_{0\to 1}\left(\overset{3}{\underset{q=0}{⨂}}R_{z}^{\left(q\right)}\left(\phi_{\ell q}\right)R_{y}^{\left(q\right)}\left(\theta_{\ell q}\right)\right)\right].$$

Here the subscript “ns” means that the circuit is used to generate a non-symmetric state. In the numerical experiment, the circuit depth is fixed as L=3. The index ℓ=1,2,3 labels the circuit layer, and the index q=0,1,2,3 labels the four qubits. For each layer *ℓ* and each qubit *q*, the rotation parameters $\theta_{\ell q}$ and $\phi_{\ell q}$ are sampled independently from the uniform distribution on [0,2π). The random seed is fixed before sampling, and after this single random draw all the parameters are kept unchanged throughout the scan of the damping coefficient γ. Therefore, the only varying parameter in this state family is the damping coefficient γ. The single-qubit gates $R_{y}^{\left(q\right)}\left(\theta_{\ell q}\right)$ and $R_{z}^{\left(q\right)}\left(\phi_{\ell q}\right)$ denote rotations acting on the *q*-th qubit. Their matrix forms are

$$R_{y}\left(\theta \right)=\left(\begin{array}{cc} \cos\frac{\theta }{2} & -\sin\frac{\theta }{2}\\ \sin\frac{\theta }{2} & \cos\frac{\theta }{2} \end{array}\right),\;R_{z}\left(\phi \right)=\left(\begin{array}{cc} e^{-i\phi /2} & 0\\ 0 & e^{i\phi /2} \end{array}\right).$$

The superscript *q* means that the corresponding one-qubit gate is embedded into the four-qubit Hilbert space and acts nontrivially only on the *q*-th qubit. Namely,

$$R_{a}^{\left(q\right)}\left(\alpha \right)=I_{2}^{⊗q}⊗R_{a}\left(\alpha \right)⊗I_{2}^{⊗(3-q)},\;a\in \left\{y,z\right\},$$

where $I_{2}$ is the 2×2 identity matrix. The operator $C_{q\to q+1}$ denotes the CNOT gate with qubit *q* as the control qubit and qubit q+1 as the target qubit. In the computational basis, it maps $|x_{q},x_{q+1}\rangle ⟼|x_{q},x_{q+1}⊕x_{q}\rangle ,$ where $x_{q},x_{q+1}\in \left\{0,1\right\}$ and ⊕ denotes addition modulo 2. Thus, each layer of the circuit consists of local single-qubit rotations followed by an ordered nearest-neighbor CNOT chain 0→1→2→3. This produces the non-symmetric pure state

$$|\psi_{\mathrm{ns}}\rangle =U_{\mathrm{ns}}|0000\rangle ,\;\rho_{0}=|\psi_{\mathrm{ns}}\rangle \langle \psi_{\mathrm{ns}}|.$$

Second, we introduce local energy-relaxation noise by applying the same amplitude-damping channel independently to each qubit. For a single qubit, the amplitude-damping channel with damping coefficient γ∈[0,1] is given by

$$E_{\gamma }^{\mathrm{AD}}\left(\rho \right)=K_{0}\rho K_{0}^{†}+K_{1}\rho K_{1}^{†},$$

where the Kraus operators are

$$K_{0}=\left(\begin{array}{cc} 1 & 0\\ 0 & \sqrt{1-\gamma } \end{array}\right),\;K_{1}=\left(\begin{array}{cc} 0 & \sqrt{\gamma }\\ 0 & 0 \end{array}\right).$$

The parameter γ describes the strength of the local amplitude damping. When γ=0, the channel reduces to the identity channel. When γ approaches one, the excited-state component is strongly relaxed toward the ground state. Applying this channel independently to all four qubits gives the noisy circuit-generated state family

(29) $$\rho_{\mathrm{AD}}\left(\gamma \right)={\left(E_{\gamma }^{\mathrm{AD}}\right)}^{⊗4}\left(U_{\mathrm{ns}}\left|0000\rangle \langle 0000\right|U_{\mathrm{ns}}^{†}\right),\;\gamma \in \left[0,1\right].$$

Once the random circuit $U_{\mathrm{ns}}$ is fixed, varying γ generates a continuous one-parameter family of noisy circuit states. In the numerical experiment, we sample 200 values of γ from [0,1], namely $\gamma_{j}=\frac{j-1}{199},j=1,\ldots ,200,$ and obtain the corresponding states ${\left\{\rho_{\mathrm{AD}}\left(\gamma_{j}\right)\right\}}_{j=1}^{200}.$ These states are neither Werner states nor random density matrices generated by the normalization $\frac{A^{†}A}{\mathrm{Tr}(A^{†}A)}$. Instead, they retain a clear circuit-and-noise structure: the initial state is prepared by a finite-depth parameterized quantum circuit, while the subsequent evolution is generated by local amplitude-damping noise. Hence, this family provides a more physically interpretable test case for evaluating whether the learned surrogate model can generalize beyond the random-matrix training distribution and the highly symmetric Werner state benchmark.

**Figure 6:**
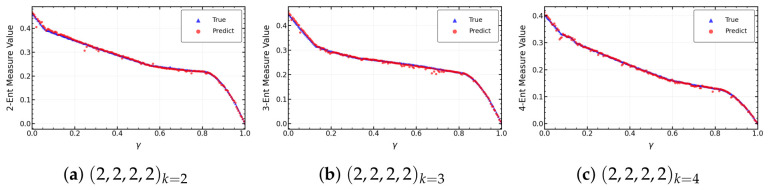
Comparison between the true values ${\tilde{E}}_{w,4}^{\left(k,4\right)}\left(\rho_{\mathrm{AD}}\left(\gamma \right)\right)$ and the predicted values on the 4-qubit noisy circuit-generated states.

Figure 6 presents the numerical results for this noisy circuit-generated family. The horizontal axis denotes the damping coefficient γ, and the vertical axis denotes the corresponding witness-based *k*-entanglement value. The blue markers represent the reference values obtained by the direct evaluation method, while the red markers represent the predictions given by the learned ensemble model. As γ increases, the entanglement value shows an overall decreasing tendency, which is consistent with the progressive suppression of quantum correlations under local amplitude-damping noise. More importantly, the predicted values closely follow the reference values over the whole damping range. The model captures not only the relatively smooth decay in the intermediate region, but also the sharp decrease in the strong-damping regime where the entanglement value rapidly approaches zero. Only small local deviations are observed, and no evident systematic bias appears in the plotted results.

This additional test shows that the learned model is not merely fitted to the random-matrix training distribution or calibrated on the highly symmetric Werner state family. It can also provide accurate surrogate predictions for a structured noisy state family generated by a physically motivated circuit-and-noise process. The inference time remains at the millisecond level for these four-qubit states, indicating that the computational efficiency of the surrogate model is retained in this additional test.

Overall, the hierarchical ensemble framework provides an efficient surrogate model for estimating witness-based *k*-entanglement quantities. Once trained, the model enables rapid inference directly from density matrices and substantially reduces the computational cost compared with direct optimization-based evaluation. The numerical results on Werner states and noisy circuit-generated states show that the learned mapping achieves good approximation accuracy within the tested system sizes and state families. This framework therefore provides a practical connection between data-driven approximation and quantum-state characterization, while further validation is still needed for larger systems, broader classes of physically relevant states, and experimental data affected by reconstruction noise.

## 5. Conclusions

This work addresses the high computational cost and real-time evaluation of strict *k*-entanglement quantification in multipartite quantum systems by developing a general machine learning framework. By reformulating the computation of $E_{w}^{\left(k,n\right)}$ as a supervised regression task, the framework transforms an originally exponential-scale procedure, which relies on high-dimensional optimization and exhaustive subsystem searches, into a single forward pass, substantially reducing computation time.

A heterogeneous ensemble of MLP, CNN, and LightGBM models captures complementary algorithmic biases and achieves a balance between efficiency and numerical stability. Numerical experiments are presented for the cases n=3 and n=4 as representative demonstrations of the proposed framework. The framework itself is formulated for general *n*-partite systems, while its practical extension to larger systems is mainly limited by the cost of constructing reliable witness-based training labels rather than by the inference stage of the learning model. The results demonstrate millisecond-level inference per quantum state and show more than five orders of magnitude speed-up compared with the software tool in [21], while maintaining accuracy consistent with the witness-based numerical values. Predictions for Werner states further show that the ensemble reproduces the expected theoretical behavior within the tested configurations. In addition, a four-qubit noisy circuit-generated state family, obtained from finite-depth circuit preparation followed by local amplitude-damping noise, is included as a structured physical test beyond random density matrices and Werner states. The results indicate that the trained ensemble can also track the witness-based *k*-entanglement variation for this physically motivated noisy state family within the tested parameter range.

The proposed framework bridges the gap between rigorous theoretical quantification and efficient numerical evaluation. Its efficiency suggests potential applications in online entanglement monitoring, adaptive feedback control, and large-scale parameter sweeps. It should also be noted that the present MLP, CNN, and LightGBM models do not explicitly enforce subsystem permutation invariance. Designing permutation-invariant or permutation-equivariant models, such as DeepSets, graph neural networks, permutation-equivariant neural networks, or symmetry-aware transformer architectures, is an important direction for future work. Future work may also extend this approach to other forms of quantum correlations, including quantum discord and multipartite mutual information, and explore robust learning under partial or noisy observations to better align with realistic laboratory conditions.

## Figures and Tables

**Table 1:** The MAE values of different machine learning models trained on nine datasets.

MAE ($\times 10^{-3}$)	Model			
$\delta_{k}$	MLP	CNN	LightGBM	Ensemble
${\left(2,4\right)}_{k=2}$	0.740	0.700	9.701	0.492
${\left(2,8\right)}_{k=2}$	1.914	1.877	6.352	1.747
${\left(2,2,2\right)}_{k=2}$	1.875	1.854	5.445	1.518
${\left(2,2,2\right)}_{k=3}$	2.429	2.243	5.792	1.957
${\left(2,4,2\right)}_{k=2}$	1.550	1.380	5.241	1.352
${\left(2,4,2\right)}_{k=3}$	1.681	1.550	5.475	1.410
${\left(2,2,2,2\right)}_{k=2}$	1.438	1.241	5.745	1.930
${\left(2,2,2,2\right)}_{k=3}$	1.203	1.057	5.540	1.452
${\left(2,2,2,2\right)}_{k=4}$	1.464	1.410	5.983	1.769

**Table 2:** The MSE values of different machine learning models trained on nine datasets.

MSE ($\times 10^{-5}$)	Model			
$\delta_{k}$	MLP	CNN	LightGBM	Ensemble
${\left(2,4\right)}_{k=2}$	1.910	1.870	7.570	0.890
${\left(2,8\right)}_{k=2}$	1.841	1.860	8.612	0.920
${\left(2,2,2\right)}_{k=2}$	0.700	0.702	5.121	0.500
${\left(2,2,2\right)}_{k=3}$	1.114	1.105	5.610	0.764
${\left(2,4,2\right)}_{k=2}$	1.935	1.907	7.837	0.910
${\left(2,4,2\right)}_{k=3}$	1.811	1.776	8.011	0.882
${\left(2,2,2,2\right)}_{k=2}$	1.731	1.713	8.55	0.901
${\left(2,2,2,2\right)}_{k=3}$	1.862	1.892	8.601	0.892
${\left(2,2,2,2\right)}_{k=4}$	1.821	1.800	7.232	0.904

**Table 3:** The $R^{2}$ scores of different machine learning models trained on nine datasets.

$R^{2}$	Model			
$\delta_{k}$	MLP	CNN	LightGBM	Ensemble
${\left(2,4\right)}_{k=2}$	0.990	0.991	0.875	0.992
${\left(2,8\right)}_{k=2}$	0.988	0.989	0.887	0.990
${\left(2,2,2\right)}_{k=2}$	0.990	0.990	0.923	0.993
${\left(2,2,2\right)}_{k=3}$	0.983	0.985	0.911	0.988
${\left(2,4,2\right)}_{k=2}$	0.989	0.990	0.891	0.991
${\left(2,4,2\right)}_{k=3}$	0.987	0.988	0.872	0.989
${\left(2,2,2,2\right)}_{k=2}$	0.990	0.991	0.883	0.991
${\left(2,2,2,2\right)}_{k=3}$	0.989	0.990	0.871	0.990
${\left(2,2,2,2\right)}_{k=4}$	0.988	0.990	0.886	0.990

**Table 4:** The time(s) of different machine learning models trained on nine datasets.

Time(s)	Model			
$\delta_{k}$	MLP	CNN	LightGBM	Ensemble
${\left(2,4\right)}_{k=2}$	$1.917\times 10^{-4}$	$1.673\times 10^{-4}$	$8.565\times 10^{-5}$	$4.877\times 10^{-4}$
${\left(2,8\right)}_{k=2}$	$1.294\times 10^{-4}$	$1.188\times 10^{-4}$	$9.022\times 10^{-5}$	$4.003\times 10^{-4}$
${\left(2,2,2\right)}_{k=2}$	$1.821\times 10^{-4}$	$1.695\times 10^{-4}$	$9.221\times 10^{-5}$	$4.153\times 10^{-4}$
${\left(2,2,2\right)}_{k=3}$	$1.911\times 10^{-4}$	$1.902\times 10^{-4}$	$9.002\times 10^{-5}$	$4.032\times 10^{-4}$
${\left(2,4,2\right)}_{k=2}$	$2.047\times 10^{-4}$	$1.985\times 10^{-4}$	$8.936\times 10^{-5}$	$4.966\times 10^{-4}$
${\left(2,4,2\right)}_{k=3}$	$1.983\times 10^{-4}$	$1.677\times 10^{-4}$	$9.439\times 10^{-5}$	$4.825\times 10^{-4}$
${\left(2,2,2,2\right)}_{k=2}$	$1.511\times 10^{-4}$	$1.237\times 10^{-4}$	$8.594\times 10^{-5}$	$3.983\times 10^{-4}$
${\left(2,2,2,2\right)}_{k=3}$	$1.619\times 10^{-4}$	$1.004\times 10^{-4}$	$9.059\times 10^{-5}$	$3.534\times 10^{-4}$
${\left(2,2,2,2\right)}_{k=4}$	$1.877\times 10^{-4}$	$1.354\times 10^{-4}$	$9.411\times 10^{-5}$	$4.196\times 10^{-4}$

**Table 5:** Comparison of inference time(s) for Werner states between the final ensemble ML model and the software in [21].

System Configuration	Time(s)	
$\delta_{k}$	Ensemble ML model	Software in [21]
${\left(2,2,2\right)}_{k=2}$	$4.702\times 10^{-4}$	48.753
${\left(2,2,2\right)}_{k=3}$	$4.898\times 10^{-4}$	47.597
${\left(2,2,2,2\right)}_{k=2}$	$4.868\times 10^{-4}$	165.725
${\left(2,2,2,2\right)}_{k=3}$	$4.628\times 10^{-4}$	171.285
${\left(2,2,2,2\right)}_{k=4}$	$4.645\times 10^{-4}$	166.754

## Data Availability

The data presented in this study are openly available at https://github.com/GuoJie1112/Data-Driven-Quantification-of-Quantum-k--Entanglement-via-Machine-Learning (accessed on 14 July 2026).
